# Characterization of pneumococcal serotype 7F in vaccine conjugation

**DOI:** 10.1007/s10719-023-10125-8

**Published:** 2023-07-04

**Authors:** James Z Deng, Xiujuan Jia, Chengli Zong, Jian He, Sha Ha, Ping Zhuang

**Affiliations:** 1 Vaccine Analytical Research & Development, 770 Sumneytown Pike, P. O. Box 4, West Point, PA WP46-3305, 19486 USA; 2 Small Molecule Analytical Research & Development, Analytical Research & Development Merck & Co., Inc, Rahway, NJ USA

**Keywords:** Pneumococcal conjugate vaccine, Pneumococcal capsular polysaccharide 7F, Ultra-performance liquid chromatography (UPLC), Size-exclusion chromatography (SEC), Multi-angle light scattering (MALS), Dynamic light scattering (DLS)

## Abstract

*Streptococcus pneumoniae* is a highly invasive bacterial pathogen that can cause a range of illnesses. Pneumococcal capsular polysaccharides (CPS) are the main virulence factors that causes invasive pneumococcal disease (IPD). Pneumococcal CPS serotype 7F along with a few other serotypes is more invasive and likely to cause IPD. Therefore, 7F is a target for pneumococcal vaccine development, and is included in the two recently approved multi-valent pneumococcal conjugated vaccines, i.e. VAXNEUVANCE and PREVNAR 20.

To support process and development of our 15-valent pneumococcal conjugated vaccine (PCV15), chromatographic methods have been developed for 7F polysaccharide and conjugate characterization. A size-exclusion chromatography (SEC) method with UV, light scattering and refractive index detections was employed for concentration, size and conformation analysis. A reversed-phase ultra-performance liquid chromatography (RP-UPLC) method was used for analysis of conjugate monosaccharide composition and degree of conjugation. The collective information obtained by these chromatographic analysis provided insights into the pneumococcal conjugate and conjugation process.

**Graphical Abstract:**

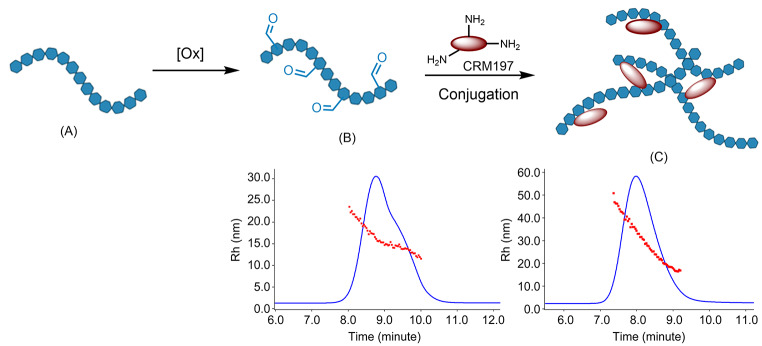

**Supplementary Information:**

The online version contains supplementary material available at 10.1007/s10719-023-10125-8.

## Introduction

*Streptococcus pneumoniae* (*S. pneumoniae*) has been identified to associate with a range of serious illnesses, such as pneumonia, meningitis, otitis media and other invasive pneumococcal diseases (IPD). Capsular polysaccharides (CPS) from *S. pneumoniae* are known to be immunogenic. By far, over a hundred pneumococcal CPS serotypes have been identified, among which more than 30 serotypes can cause streptococcus infections [1, 2]. Initially, multi-valent pneumococcal polysaccharide vaccine (PPSV), such as 23-valent PNEUMOVAX 23 was developed with success in adults [3]. However, polysaccharide based vaccines were less immunogenic for infants whose immune system is not fully developed and incapable of developing long-lasting immunity against pneumococcal infections. Pneumococcal conjugate vaccines (PCVs) were developed to recruit helper T cells, and offer long lasting and more robust T-cell dependent immune responses and enhanced immunological memory. In PCVs, e.g. the recent FDA approved VAXNEUVANCE or PREVNAR 20, each polysaccharide serotype is modified and conjugated to a carrier protein, such as the diphtheria toxin mutant CRM197 (Cross-Reactive-Material-197) [4–6].

Serotype 7F is among the serotypes with high invasive capacity and propensity to cause invasive diseases [7, 8]. Vaccines with 7F conjugate not only provide protection for the specific target serotype, but also could elicit protective immune response against other untargeted serotypes, such as 7A [9]. Therefore, serotype 7F conjugate is desired for novel PCV development [10]. In our effort to develop a 15-valent pneumococcal conjugate vaccine (PCV15), serotype 7F was formulated with 1, 3, 4, 5, 6A, 6B, 9V, 14, 18C, 19A, 19F, 22F, 23F, 33F and an aluminum adjuvant. Extensive analytical characterizations are required for 7F polysaccharide and conjugate, as well as for each other serotypes. Here, we demonstrate our chromatographic analytical strategies for 7F. These strategies can be easily expanded to other PCV serotypes.

**Fig. 1:**
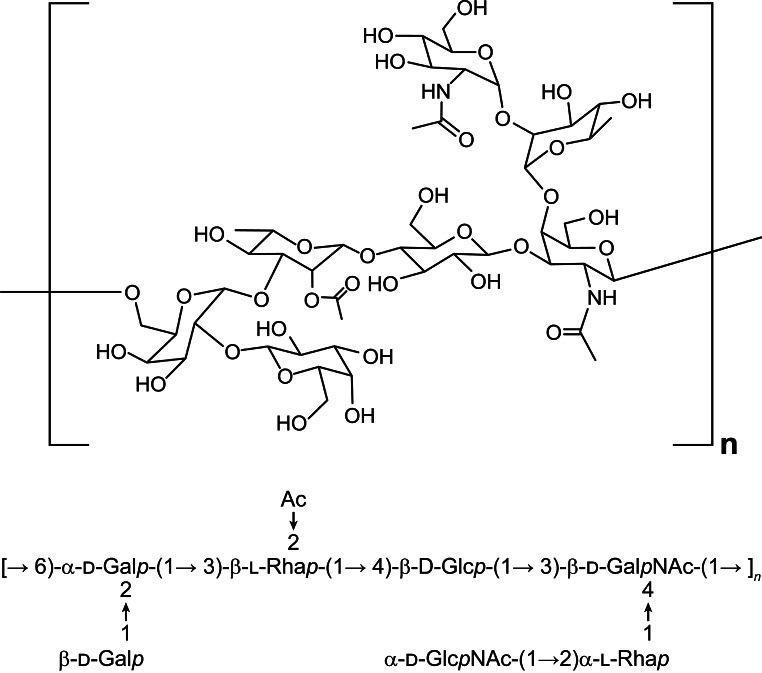
The structure of pneumococcal 7F polysaccharide. Monosaccharide sequence that forms a 7F polysaccharide repeating unit is shown below the structure

**Fig. 2:**
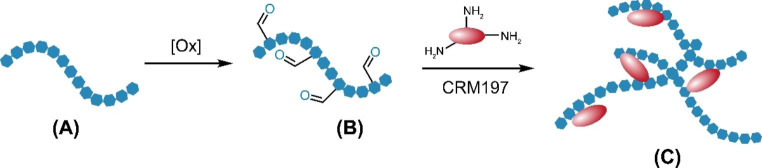
Activation and conjugation of pneumococcal 7F polysaccharide. The polysaccharide (**A**) was activated to aldehyde containing species (**B**) by oxidation, before conjugation with CRM197 protein to form pneumococcal conjugate (**C**)

In a 7F capsular polysaccharide, each repeating unit (RU) is formed by seven monosaccharides comprised of two Galactoses (Gal), one N-Acetyl galactosamine (GalNAc), one N-Acetylglucosamine (GlcNAc), one Glucose (Glc) and two Rhamnose (Rha) species (Fig. 1). In our conjugation process, it was first converted into aldehyde bearing polysaccharide species (Fig. 2, (B)) by an oxidative cleavage reaction. The aldehyde species was conjugated to the lysine residues in CRM197 protein to form the 7F pneumococcal conjugate. The resulting PCV product is a complex cross-linked, polydisperse polymeric system (Fig. 2, (C)) that requires a plethora of analytical tools for process and quality control [11, 12].

Although the structure of 7F polysaccharide has been determined by nuclear magnetic resonance (NMR) [13, 14], analysis of the more complex polysaccharide protein conjugate by NMR and/or mass spectroscopy still meets considerable challenges. Chromatography based methods are still the major tools for pneumococcal conjugate vaccine process development and monitoring. Size-exclusion chromatography (SEC) can separate the large polysaccharide and conjugate on the column, while maintaining their native states in solution. Furthermore, when coupled with light scattering and other detectors, it can provide information about molar mass (Mw), conformation and shape of the analytes [15–18]. These information is very valuable to understand pneumococcal conjugates with Mw more than 1000 kDa, where 3-dimensional structure determination by NMR or X-ray is still challenging [19].

In this study, we connect both dynamic light scattering (DLS) and multi-angle static light scattering (MALS) detectors to the SEC, plus two concentration detectors with UV and refractive index (RI) for the SEC-UV-MALS-DLS-RI method (Sect. 2.3). In a single HPLC run, we can measure critical quality attributes (CQAs), such as molecular weight (Mw), radius of gyration (Rg), hydrodynamic radius (Rh) and conjugate protein to polysaccharide ratio (Pr/Ps), as well as the change of these attributes during process and storage. The Burchard shape factor ρ (Rg/Rh) and Burchart-Stockmayer plot is used to evaluate the shape/conformation of the 7F polysaccharide and its CRM197 conjugate [20, 21]. These provide timely and critical analytical input for process development.

**Fig. 3:**
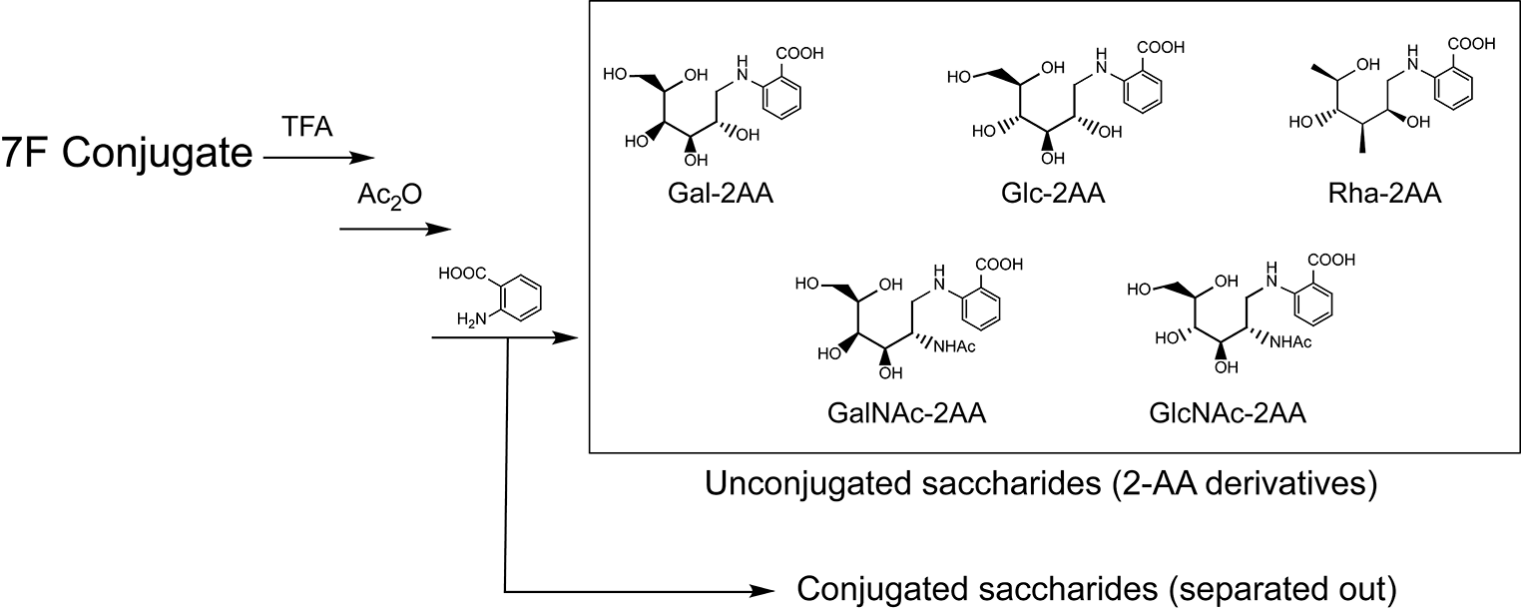
Prepare 7F conjugate for RP-UPLC analysis. A 7F conjugate was hydrolyzed to monosaccharides. Each monosaccharide was derivatized by 2-AA. The monosaccharide 2-AA derivatives were separated and analyzed on UPLC.

Besides using the SEC method for polysaccharides and conjugates, we sought to have a chromatographic tool to help us to assess the conjugation on each monosaccharide. Literature has reported quantification of unconjugated polysaccharides on high-performance anion-exchange chromatography with pulsed amperometric detection (HPAEC/PAD) [22]. A GC/MS method was also reported to quantify polysaccharides and conjugates [23]. To develop a chromatography method that can be commonly adapted in the process and quality control environment, a reversed-phase ultra-performance liquid chromatography (RP-UPLC) method was employed with sensitive fluorescence (FLR) detection (Sect. 2.5). In this, 7F conjugate was first degraded to monosaccharides by acid hydrolysis and labeled by 2-aminobenzoic acid (2-AA). The RP-UPLC method separates and quantify the labeled monosaccharide species in comparison with monosaccharide standards. The CRM197 conjugated monosaccharides will be separated out and excluded from quantification (Fig. 3). Therefore, the RP-UPLC measured concentration from each monosaccharide represent the unconjugated intact saccharide, and can be used to assess the modification/conjugation level of each monosaccharide. Combination of the SEC and UPLC analysis would provide understandings of 7F conjugate at different molecular levels.

## Materials and methods

### Commercial reagents and materials

All Chemicals and monosaccharide standards were purchased from Millipore Sigma (St. Louis, MO). Acquity UPLC BEH C18 2.1 × 100 mm column (1.7 μm, 130 Å) was obtained from Waters Corporation (Milford, MA). TSKgel GMPWxL size-exclusion chromatography column (7.8 × 300 mm, 13 μm) was ordered from Tosoh Bioscience (Japan). Bovine Serum Albumin (BSA) Standard Ampules (2 mg/mL) was ordered from Thermo Fisher (Catalog# 23,209).

### 7F polysaccharide and 7F conjugate samples

All polysaccharides, activated polysaccharides and conjugates were produced internally by our vaccine process development group.

### SEC-UV-MALS-DLS-RI analysis of 7F polysaccharides and conjugates

The size-exclusion chromatography analysis was performed on an Agilent system (1260 Infinity) (Agilent, DE, USA) equipped with UV diode array detector (DAD). A Wyatt static multi-angle static light scattering (MALS) detector (DAWN® or MiniDAWN®), a Wyatt Quasi-Elastic Light Scattering (QELS) DLS detector and an Optilab® T-rEX refractive index (RI) detector (Wyatt Technology Corp., Santa Barbara) were added in tandem after the Agilent UV detector. The detectors are in a tandem sequence of UV-MALS-DLS-RI. Peak alignment and band broadening were performed with a monodisperse BSA standard and the aligned chromatographic data are reported. All data were collected and analyzed with ASTRA software (ASTRA 7 or 8). A TSKgel GMPWxL column (7.8 × 300 mm, Tosoh Bioscience, Japan) was used with a 10 mM Bis-Tris, 150 mM NaCl, pH 6.8 mobile phase at constant flow rate of 0.8 mL/min. Column temperature was set at 35 °C with 25 min HPLC run time for each injection.

7F polysaccharide was analyzed by a standard Astra light scattering method [24] to report polysaccharide Mw and content, whereas 7F conjugate was analyzed by Astra protein conjugate method that deconvolutes protein (CRM197) attributes from polysaccharide attributes in a conjugate. This allows measurement of both protein and polysaccharide Mw and content from a single injection [25]. Zimm formalism was used for MALS to calculate weight averaged molar mass (Mw) and radius of gyration (Rg) [26]. The hydrodynamic radius (Rh) was calculated from the DLS measured translational diffusion coefficient through the Stokes-Einstein relationship [27, 28]. The UV A280 extinction coefficient for CRM197 is 0.903 mL/(mg•cm). A generic protein dn/dc value of 0.185 mL/g was used for CRM197 and BSA. A normalized dn/dc of 0.133 mL/g was used for 7F polysaccharides [29].

### ^1^ H NMR analysis of activated 7F polysaccharide intermediate

NMR sample was placed in magnet after the 7F polysaccharide powder sample was dissolved in 700 µL D_2_O (99.9 atom %D, Cambridge Isotope Laboratories). 1D proton NMR spectra of the polysaccharides were recorded on a Varian Unity Inova 600 MHz NMR spectrometer equipped with a cryogenic HCN probe. The probe temperature was set to 49 °C and standard Varian pulse sequences were used for 1D proton NMR spectra. Data were acquired with 8 kHz spectrum width, covering from − 1 to 12.0 ppm, with 0.3 Hz digital resolution. For the samples in D_2_O, transients of 4 scans were acquired following 2 steady state scans and 57 s relaxation delay. 1D proton NMR data were collected at timepoints at ambient temperature (20 ^o^C), after the 7F polysaccharide was dissolved in D_2_O, i.e. 0 and 20 h. Experimental data were processed using VnmrJ 4.2 and MestReNova. The observed chemical shifts (δ) were referenced to internal sodium 4,4-dimethyl-4-silapentane-1-sulfonate (DSS-d6) at 0.0 ppm.

### Analysis of monosaccharides in 7F pneumococcal conjugate by RP-UPLC

#### Preparation of standards and 7F sample for monosaccharide analysis

Nine monosaccharide standards from Galactoses (Gal), N-Acetyl galactosamine (GalNAc), N-Acetylglucosamine (GlcNAc), Glucose (Glc), Rhamnose (Rha), Ribose (Rib), Mannose (Man), N-Acetyl-D-Mannosamine (ManNac) and Arabinose (Ara) were mixed together in water as a 30 µg/mL stock solution. A 3 µg/mL standard solution of the nine monosaccharides was prepared by 10-fold dilution of the 30 µg/mL stock solution. The 5-point standard curve was prepared by adding 200 µL of 2 N trifluoroacetic acid (TFA) into different amount of the 9-monosaccharide standard in 5 different sealed vials (Supplementary Table S1). The TFA hydrolysis was completed by heating the vials at 100 ^o^C for 2 h in the sealed vails. After hydrolysis, the solvent was evaporated on a GenVac EZ-2 evaporator (SP Scientific). 35 µL solution of sodium carbonate, pH 9 was added into each vial, followed by 15 µL of acetic anhydride to cap the free amine groups. The vials are re-capped and incubated at ambient temperature for 30–45 min. After this, solvent in each vial was removed by GenVac again. 20 µL Mobile phase A (50 mM sodium acetate solution, pH 4.1) and 20 µL of 1.9 M anthranilic acid (2-AA, Millipore Sigma) DMSO solution was added into each vial, followed by 10 µL of 1 M sodium cyanoborohydride solution in THF (Millipore Sigma). After sealing each vial, the 2-AA labeling reaction was heated at 85 ^o^C for one hour. The reaction was cooled to ambient temperature and solution in the vial was spun down by centrifugation at 500 g for one minute. Solution in each vial was transferred into a Waters total recovery vial (Waters Corporation, Milford, MA) for HPLC analysis.

7F pneumococcal conjugate sample was diluted by water to desired concentration before analysis. 20 µL of the diluted sample was taken into the same reaction sequence above as the standards.

#### RP-UPLC analysis of hydrolyzed 7F conjugate

The RP-UPLC was performed on a Waters ACQUITY UPLC system (Waters Corporation, Milford, MA), including a quaternary pump, sample manager, column component, fluorescence (FLR) detector. The FLR was detected using excitation wavelength at 360 nm and emission wavelength at 435 nm (*Ex* 360 nm, *Em* 435 nm). Waters Acquity UPLC BEH C18 column (2.1 × 100 mm) was used, with column temperature at 40 ^o^C. The gradient elution in Table 1 was formed by Mobile phase A (50 mM sodium acetate solution, pH 4.1) and Mobile phase B (20% mobile phase A, 80% MeOH solution) with constant flow rate of 0.3 mL/min.

**Table 1:** RP-UPLC flow rate and gradient table

Time (min)	Flow rate (mL/min)	Mobile phase	
		%A	%B
0	0.3	97.0	3.0
1	0.3	97.0	3.0
5	0.3	92.5	7.5
13	0.3	92.5	7.5
28	0.3	80.0	20.0

## Results and discussion

### SEC-UV-MALS-DLS-RI characterization of serotype 7F in the conjugation process

**Fig. 4:**
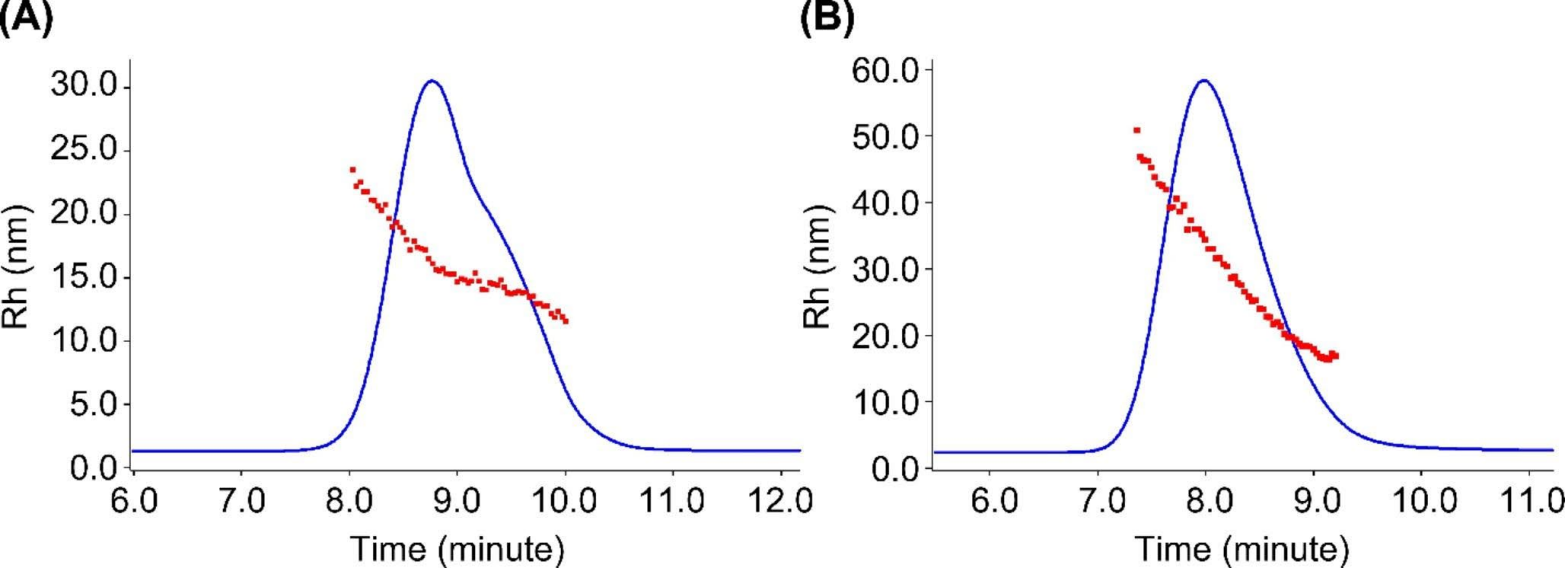
Hydrodynamic radius (Rh) distribution on SEC: (**A**) 7F polysaccharide; (**B**) 7F conjugate

The SEC-UV-MALS-DLS-RI method was used to measure critical quality attributes for both unconjugated polysaccharide intermediate and the conjugate formed in the conjugation reaction. These measured analytical attributes are listed in Table 2. During conjugation, the CRM197 protein reacted with activated 7F polysaccharide to form a 7F conjugate with Pr/Ps (w/w) ratio of 0.90. The conjugate has larger size (Mw, Rg and Rh) than the unconjugated polysaccharide starting material. The greatly increased size (more than 10-fold increase in Mw) was the result of crosslinking of polysaccharide chains by the CRM197 protein, as both CRM197 and activated 7F polysaccharide have multiple sites for conjugation. In the hydrodynamic radius (Rh) distribution plot (Fig. 4) from dynamic light scattering (DLS), the 7F polysaccharide showed two distinctive Rh distribution patterns that resulted in a shoulder peak on the SEC chromatogram. After conjugation, a smooth Rh distribution across a single conjugate peak was observed. The 7F conjugate Rh range spans from ~ 15 to 50 nm, whereas the unconjugated polysaccharide is smaller with a narrower Rh distribution (~ 12 to 25 nm). The increased Rh distribution range for conjugate on DLS is consistent with increased polydispersity (PDI, Mw/Mn) for conjugate from MALS measurement (Table 2, PDI).

**Table 2:** Comparison of 7F conjugate with 7F polysaccharide (Ps) on SEC-UV-MALS-DLS-RI

Sample	Mw (kDa)	PDI (Mw/Mn)	Rg (nm)	Rh (nm)	Shape factor ρ (Rg/Rh)	Pr/Ps (mg/mg)
7F Ps	296	1.07	23	16	1.42	N/A
7F Conjugate	4187	1.58	44	32	1.36	0.90

**Fig. 5:**
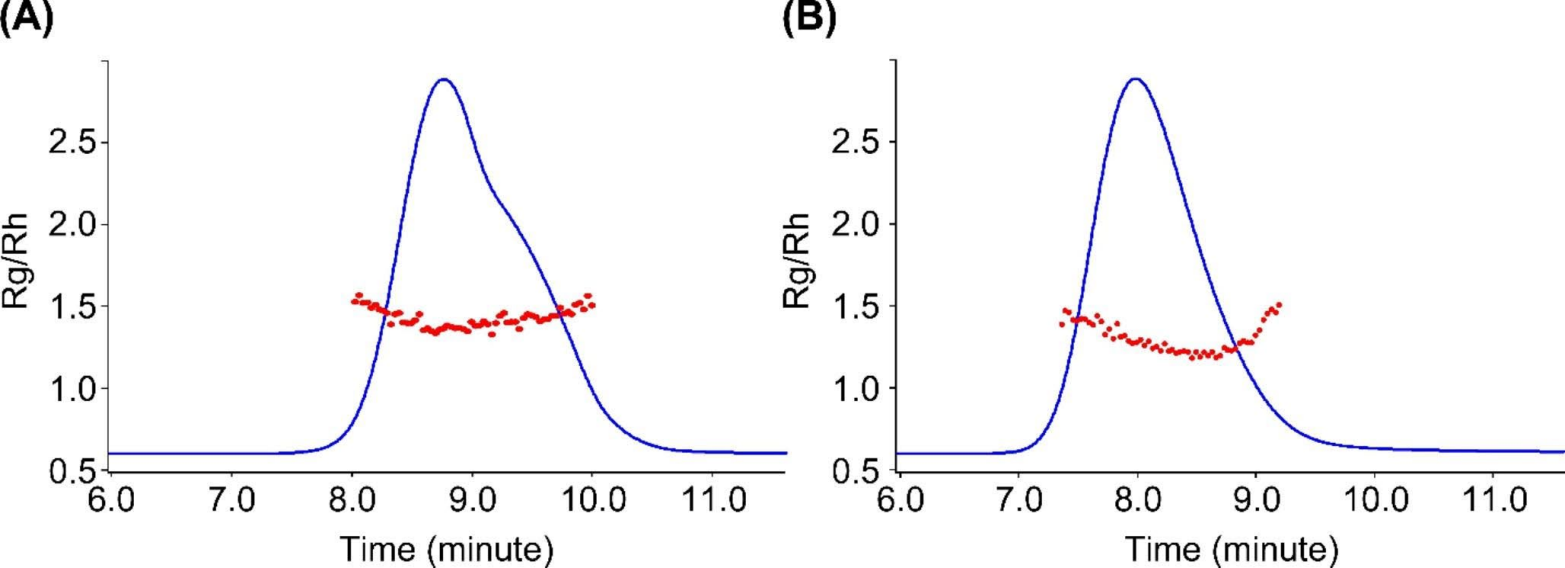
Burchard-Stockmayer plots for: (**A**) 7F polysaccharide; (**B**) 7F conjugate. Rg/Rh represents the Burchard shape factor ρ

The Burchard shape factor ρ (Rg/Rh) is an important parameter to understand polymer shape and conformations. Generally, a globular protein such as CRM197, has a ρ value of ~ 0.77, whereas the ρ value of 1.0 to 1.1 corresponds to branched chains, and 1.5 to 1.8 indicates linear coil chains [30, 31]. The overall ρ value of 1.42 for 7F polysaccharide (Tables 2 and 7F Ps) indicates that the polysaccharide adapts mostly linear coil conformations. Slightly drop of overall ρ value to 1.36 after conjugation (Tables 2 and 7F Conjugate) suggests that adding CRM197, a more compact molecule to 7F polysaccharide with more extended chain conformation, only brought in small overall conformation change. Burchard-Stockmayer plots were generated for both 7F polysaccharide and conjugate (Fig. 5). The ρ value (Rg/Rh) of 7F polysaccharide is distributed in a narrow range across the peak. This indicates that the 7F polysaccharide adopts relative consistent conformation, even though there is a shoulder peak on the chromatography. On the other hand, there is a rise of ρ value at the tail of 7F conjugate peak. This indicated there was a conformation change at the peak tail for a small population of conjugates.

### Understanding conjugate intermediate Mw change

**Fig. 6:**
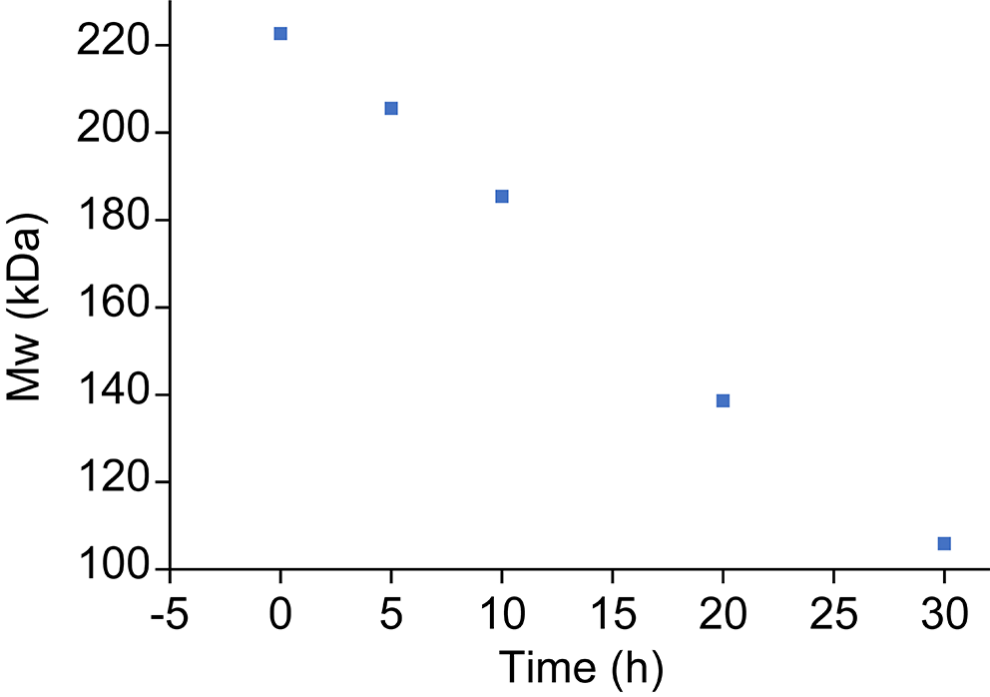
Monitoring Mw change of an activated 7F polysaccharide at 20 ^o^C

Both unconjugated 7F intermediates and 7F conjugates were stored frozen. The activated 7F polysaccharide intermediates (Ps-1 in Table 3) and 7F conjugates are stable during the standard freeze-thaw process (6 ^o^C), and their molecular weights (Mw) measured by SEC-MALS remain unchanged for seven days (Table 3). To demonstrate the ability of SEC-MALS method detect a small change in stability monitoring studies, an exploratory activated 7F polysaccharide lot was stressed at 20 ^o^C and the Mw was measured during a time course. The gradual Mw change was detected over time (Fig. 6). This highlighted the ability of SEC-MALS method to detect changes in stability study. Therefore, it can be used as a stability-indicating assay.

**Table 3:** Stability of 7F polysaccharide and conjugate in freeze-thaw at 6 ^o^C

Time in 6 ^o^C (day)	Mw (kDa)	
	Conjugate	Ps-1
0	5807	332
1	5860	337
3	5930	336
5	5884	335
7	5881	338
%RSD	0.8	0.7
Day-7/Day-0 (%)	101	102

**Fig. 7:**
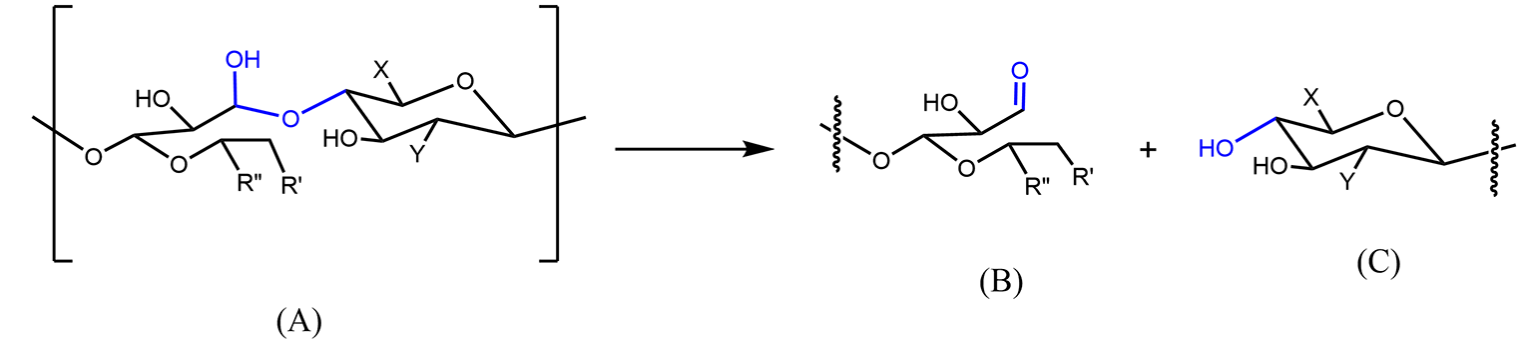
Proposed hydrolysis of activated polysaccharide structure (**A**) linked by a transitional hemiacetal bond. After hydrolysis, the original polysaccharide (**A**) was fragmented into two smaller (lower Mw) polysaccharides (**B**) and (**C**). An extra aldehyde group on (**B**) could be observed on 1 H NMR.

Considering the graduate change detected at 20 ^o^C, we hypothesized that a transition hemiacetal bond might have formed and linked polysaccharides together in the exploratory lot. Formation of such hemiacetal bond was reported in an enzymatic oxidation of polysaccharides to aldehyde species [32]. Hydrolysis of such unstable hemiacetal linkage in aqueous solution would lead to smaller polysaccharide fragments (Fig. 7), as observed by Mw decrease in Fig. 6. To further substantiate this hypothesis, the lyo powder of this activated 7F polysaccharide lot was dissolved in D_2_O and monitored by ^1^ H NMR at 0 and 20 h at ambient temperature (20 ^o^C). An extra aldehyde ^1^ H peak (~ 9.3 ppm) was observed after 20 h at ambient temperature (Supplementary Fig. S1). This suggested presence of labile precursor functional groups, such as acetal or hemiacetal in the activated polysaccharide lot. A new aldehyde functional group and fragmented polysaccharides could be formed as a result of hydrolysis of a labile acetal or hemiacetal functional group.

### Analysis of saccharide conjugation level by RP-UPLC-FLR

**Fig. 8:**
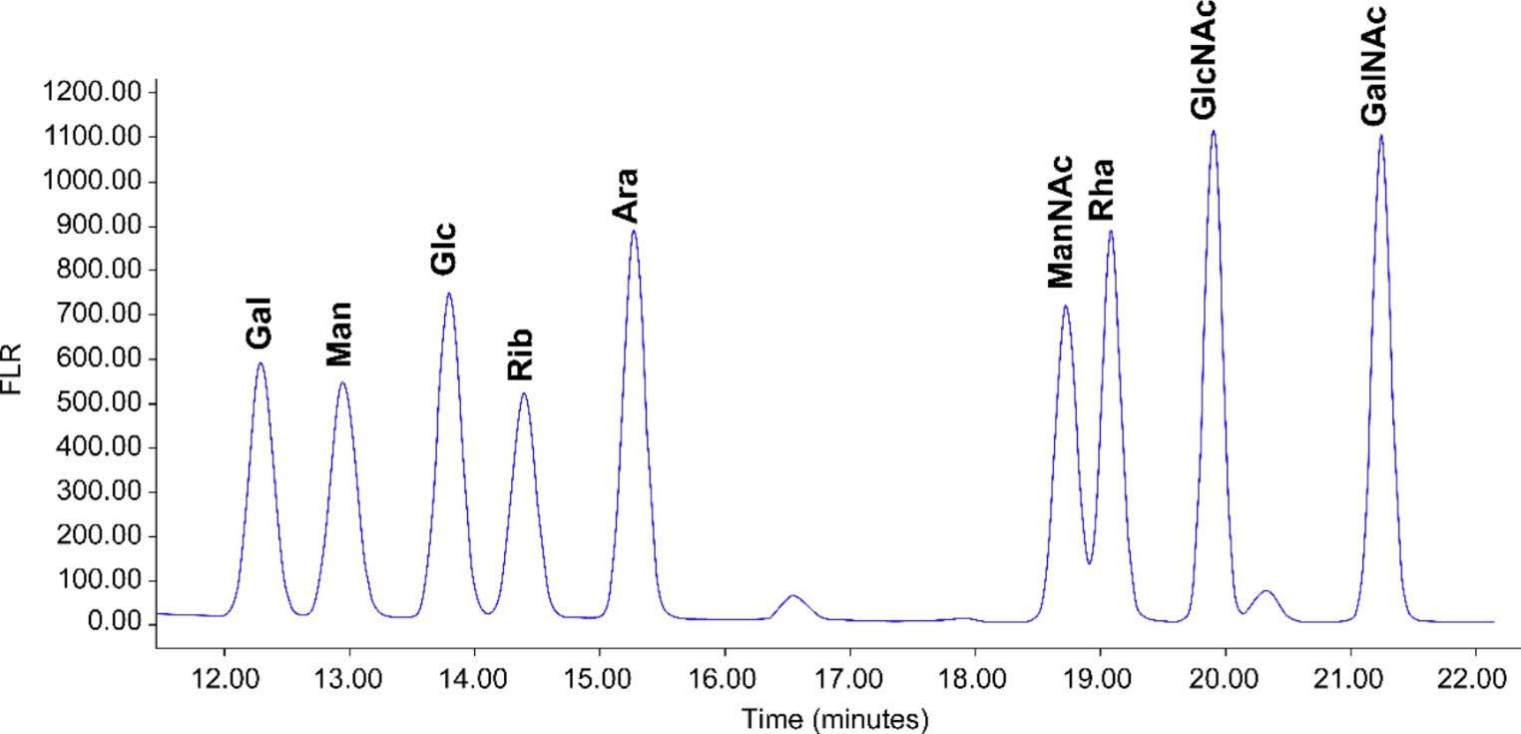
RP-UPLC separation of nine 2-AA derivatized monosaccharides

Although we have developed a powerful chromatography method that is capable of quantification of each polysaccharide in the vaccine [33], an orthogonal method to quantify each monosaccharide within a polysaccharide conjugate is desired for process understanding of the conjugation site and level within a polysaccharide repeating unit. A hydrolysis based RP-UPLC method was developed for this purpose. The RP-UPLC is capable of separating nine monosaccharide 2-AA derivatives for quantification of monosaccharides from 7F or other pneumococcal serotypes (Fig. 8 and Supplementary Fig. S2). Standard curves were generated and good linearity were observed for all 9 monosaccharides on log to log scale (Supplementary Table S2 and Fig. S3). Activation of saccharide through oxidative cleavage requires a vicinal diol as reactive site to generate aldehydes for conjugation. The GalNac saccharide in a 7F polysaccharide does not carry a diol functionality for activation and therefore could not conjugate to the CRM197 protein. The rest of the monosaccharides in the 7F i.e. Gal, Rha, Glc and GlcNac all have vicinal diols that can be activated for CRM197 conjugation (Fig. 1). Based on this structural information, the GalNac molar concentration can be used to represent the concentration for one intact monosaccharide in 7F. The rest monosaccharides were normalized against GalNac by molar concentration to assess their conjugation levels (Supplementary Table S3 and Table 5). As demonstrated in Tables 4 and 7F conjugation mostly occurred on the Galactoses (Gal) in a repeating unit, with ~ 26% of Gal conjugated. With this, we have gained a quick readout about the conjugation level and site without involving extensive structural characterization of the complex conjugate molecules.

**Table 4:** Conjugation levels on each monosaccharide

Monosaccharide	Gal	Glc	Rha	GlcNac	GalNac
[Monosaccharide] (mM)	0.578	0.432	0.761	0.403	0.391
Monosaccharide unit normalized against GalNac (one unit)	1.48	1.11	1.95	1.03	1.00
Theoretical monosaccharide unit in 7F a repeating unit	2	1	2	1	1
% intact (unconjugated) saccharide	74	111	97	103	100
% conjugation per saccharide (conjugation level)	26	0	3	0	0

## Conclusions

Pneumococcal conjugates are complex glycoconjugates with high polydispersity and heterogeneity. Development of a multivalent pneumococcal conjugate vaccine requires extensive analysis and good quality control for each individual serotype. Pneumococcal serotype 7F is among the few serotypes that have highest invasive disease potential and greatest impact on vaccine efficacy. It has a relatively more complex repeating unit structure than other pneumococcal serotypes. Using serotype 7F as an example, we have developed two orthogonal chromatographic methods as powerful tools for understanding the glycoconjugation process and for glycoconjugate analysis. Such analytical methods and strategy should find their applications in other glycoconjugates, and glycoconjugate based vaccines and biologic products.

### Electronic supplementary material

Below is the link to the electronic supplementary material.

Supplementary Material 1

## Data Availability

The datasets generated and/or analyzed during the current study are available from the corresponding author on reasonable request.

## References

[1] Geno KA, Gilbert GL, Song JY, Skovsted IC, Klugman KP, Jones C, Nahm MH (2015). Pneumococcal capsules and their types: Past, present, and future. Clin. Microbiol. Rev.

[2] Weiser JN, Ferreira DM, Paton JC (2018). Streptococcus pneumoniae: Transmission, colonization and invasion. Nat. Rev. Microbiol.

[3] Niederman MS, Folaranmib T, Buchwald UK, Musey L, Cripps AW, Johnson KD (2021). Efficacy and effectiveness of a 23-valent polysaccharide vaccine against invasive and noninvasive pneumococcal disease and related outcomes: A review of available evidence. Expert Rev. Vaccines.

[4] Micoli F, Romano MR, Carboni F (2023). Strengths and weaknesses of pneumococcal conjugate vaccines. Glycoconj. J.

[5] Greenberg D, Hoover PA, Vesikari T, Peltier C, Hurley DC, McFetridge RD, Dallas M (2018). Safety and immunogenicity of 15-valent pneumococcal conjugate vaccine (PCV15) in healthy infants. Vaccine.

[6] Cannon K, Elder C, Young M (2021). A trial to evaluate the safety and immunogenicity of a 20-valent pneumococcal conjugate vaccine in populations of adults ≥ 65 years of age with different prior pneumococcal vaccination. Vaccine.

[7] Del Amo E, Brotons P, Monsonis M, Triviño M, Iñigo M, Selva L, Sa-Leão R, Muñoz-Almagro C (2014). High invasiveness of pneumococcal serotypes included in the New Generation of Conjugate Vaccines. Clin. Microbiol. Infect.

[8] Aguiar SI, Brito MJ, Gonçalo-Marques J, Melo-Cristino J, Ramirez M (2010). Serotypes 1, 7F and 19A became the leading causes of Pediatric Invasive Pneumococcal Infections in Portugal after 7 years of Heptavalent Conjugate Vaccine Use. Vaccine.

[9] Cooper, D., Yu, X., Sidhu, M., Nahm, M.H., Fernsten, P., Jansen, K.U., Vaccine: 29(41), 7207–7211 (2011) 10.1016/j.vaccine.2011.06.056 PMC3170457 21689707

[10] Menova P, Sella M, Sellrie K, Pereira CL, Seeberger PH (2018). Identification of the minimal glycotope of Streptococcus Pneumoniae 7F capsular polysaccharide using synthetic oligosaccharides. Chem.---Eur. J.

[11] Frasch CE (2009). Preparation of bacterial polysaccharide–protein conjugates: Analytical and manufacturing challenges. Vaccine.

[12] Biemans R, Micoli F, Romano MR (2020). Glycoconjugate vaccines, production and characterization. Recent. Trends in Carbohydrate Chemistry.

[13] Moreau M, Richards JC, Perry MB, Kniskern PJ (1988). Application of high-resolution N.M.R. spectroscopy to the elucidation of the structure of the specific capsular polysaccharide of Streptococcus pneumoniae type 7F. Carbohydr. Res.

[14] Abeygunawardana C, Williams TC, Sumner JS, Hennessey JP (2000). Development and validation of an NMR-based identity assay for bacterial polysaccharides. Anal. Biochem.

[15] Podzimek S (2014). Truths and myths about the determination of Molar Mass distribution of synthetic and natural polymers by size exclusion chromatography. J. Appl. Polym. Sci.

[16] Bednar B, Hennessey JP (1993). Molecular size analysis of capsular polysaccharide preparations from Streptococcus pneumoniae. Carbohydr. Res.

[17] Deng JZ, Lancaster C, Winters MA, Phillips KM, Zhuang P, Ha S (2022). Multi-attribute characterization of pneumococcal conjugate vaccine by size-exclusion chromatography coupled with UV-MALS-RI detections. Vaccine.

[18] Deng JZ, Lin J, Chen M, Lancaster C, Zhuang P (2022). Characterization of high Molecular Weight Pneumococcal Conjugate by SEC-MALS and AF4-MALS. Polymers.

[19] Mathieu-Gaedke, M., Böker, A., Glebe, U.: How to characterize the protein structure and polymer conformation in protein-polymer conjugates – a perspective. Macromol. Chem. Phys., 2200353 (2023)

[20] Burchard W, Schmidt M, Stockmayer WH (1980). Information on Polydispersity and branching from combined quasi-elastic and Integrated Scattering. Macromolecules.

[21] Burchard W (1999). Solution Properties of branched macromolecules. Adv. Polym. Sci.

[22] Lei QP, Lamb DH, Heller R, Pietrobon P (2000). Quantitation of low level unconjugated polysaccharide in tetanus toxoid-conjugate vaccine by HPAEC/PAD following rapid separation by deoxycholate/HCl. J. Pharm. Biomed. Anal.

[23] Kim JS, Laskowich ER, Arumugham RG, Kaiser RE, MacMichael GJ (2005). Determination of saccharide content in pneumococcal polysaccharides and conjugate vaccines by GC-MSD. Anal. Biochem.

[24] Wyatt PJ (1993). Light scattering and the Absolute characterization of Macromolecules. Anal. Chim. Acta.

[25] Kendrick BS, Kerwin BA, Chang BS, Philo JS (2001). Online size-exclusion high-performance liquid Chromatography Light Scattering and Differential Refractometry Methods to determine degree of polymer conjugation to proteins and protein-protein or protein-ligand Association States. Anal. Biochem.

[26] Zimm BH (1948). The scattering of light and the Radial distribution function of high polymer solutions. J. Chem. Phys.

[27] Einstein A (1905). Über die von der molekularkinetischen Theorie der Wärme geforderte Bewegung von in ruhenden Flüssigkeiten suspendierten Teilchen. Ann. Phys.

[28] Miller CC (1924). The Stokes-Einstein law for diffusion in solution. Proc. Royal Soc. Lond. Ser. A.

[29] Theisen, A., Johann, C., Deacon, M.P., Harding, S.E.: Refractive Increment Data-Book. Nottingham University Press (2000)

[30] Kang D, Cai Z, Wei Y, Zhang H (2017). Structure and chain conformation characteristics of high acyl gellan gum polysaccharide in DMSO with sodium nitrate. Polymer.

[31] Konishi T, Yoshizaki T, Yamakawa H (1991). On the “Universal Constants” r and F of flexible polymers. Macromolecules.

[32] Mikkonen KS, Parikka K, Suuronen J, Ghafar A, Serimaa R, Tenkanen M (2014). Enzymatic oxidation as a potential new route to produce polysaccharide aerogels. RSC Adv.

[33] Deng JZ, Kuster N, Drumheller A, Lin M (2023). Antibody enhanced HPLC for serotype-specific quantitation of polysaccharides in pneumococcal conjugate vaccine. NPJ Vaccines.

