# Supplementary material for: Characterization of pneumococcal serotype 7F in vaccine conjugation
Source: Glycoconj J. 2023 Jul 4;40(5):565–73. doi: 10.1007/s10719-023-10125-8 (PMC10638203; doi:10.1007/s10719-023-10125-8)
Supplement: Supplementary file 1 — Supplementary Material 1 [file 10719_2023_10125_MOESM1_ESM.docx]

Supplementary Material for “Characterization of Pneumococcal Serotype 7F in Vaccine Conjugation”

James Z Deng*, Xiujuan Jia, Chengli Zong, Jian He, Sha Ha, Ping Zhuang

Vaccine Analytical Research & Development; Small Molecule Analytical Research & Development; Analytical Research & Development; Merck & Co., Inc., Rahway, NJ, USA

**Fig. S1. 1H NMR monitoring an activated 7F polysaccharide at 0 and 20 hour after dissolved in D_2_O. An extra aldehyde proton peak was observed between 9.2 and 9.4 ppm.**


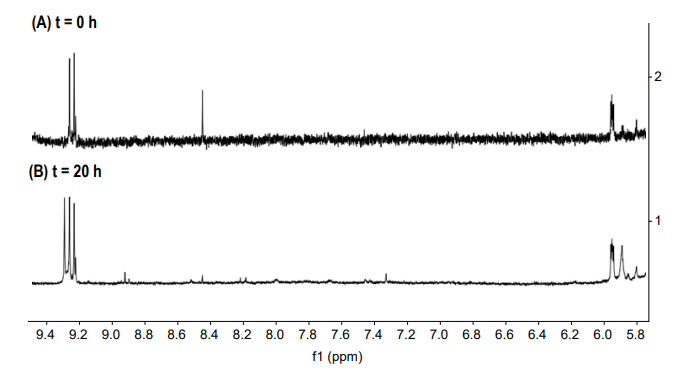


**Fig. S2. Overlay of the 2-AA derivatized monosaccharides (trace in** **blue color) from hydrolysis of 7F conjugate with nine 2-AA derivatized nine monosaccharide standards (trace in** **marmalade color) on RP-UPLC.**


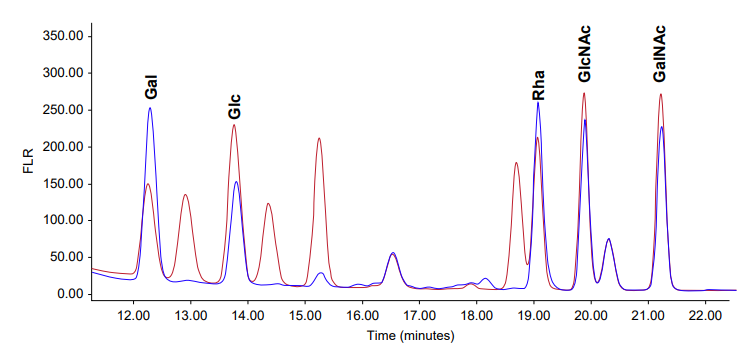


**Fig. S3. Overlay of standard injections at five concentration levels.**


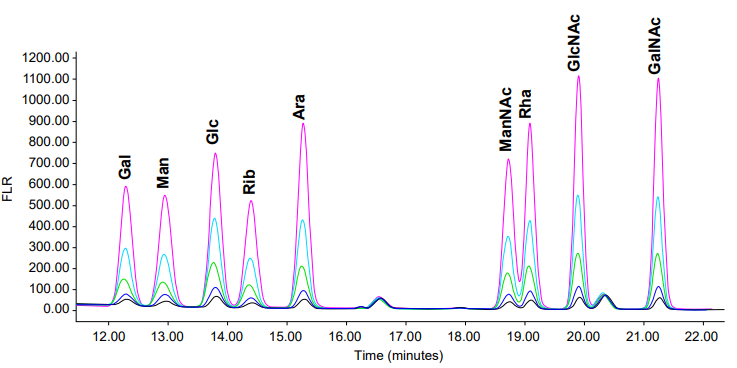


**Table S1. Sample concentrations during freeze-thraw at 6 ^o^C. All sample concentrations were maintained during freeze-thraw.**

| Day | [7F Conjugate] (mg/mL) | [Ps-1] (mg/mL) |
| --- | --- | --- |
| 0 | 3.11 | 12.1 |
| 1 | 3.12 | 12.1 |
| 3 | 3.14 | 12.0 |
| 5 | 3.11 | 11.8 |
| 7 | 3.12 | 11.7 |
| %RSD | 0.36 | 1.5 |
| Day-7/Day-0 (%) | 100 | 96 |

**Table S2. Linearity of standard curves for the nine monosaccharide standards in Log to Log scale.**

| Mono- saccharide in reaction (µg) | Log(Mono-saccharide) | Log (FLR peak area) | | | | | | | | |
| --- | --- | --- | --- | --- | --- | --- | --- | --- | --- | --- |
|  |  | Gal | Man | Glc | Rib | Ara | ManNac | Rha | GlcNac | GalNac |
| 0.06 | 0.0792 | 6.020 | 6.260 | 6.503 | 6.438 | 6.789 | 6.804 | 6.867 | 7.113 | 6.975 |
| 0.12 | 0.3802 | 6.182 | 6.728 | 6.962 | 6.746 | 7.098 | 7.092 | 7.132 | 7.252 | 7.229 |
| 0.3 | 0.7782 | 7.100 | 7.252 | 7.287 | 7.185 | 7.509 | 7.506 | 7.534 | 7.630 | 7.615 |
| 0.6 | 1.0792 | 7.485 | 7.589 | 7.634 | 7.536 | 7.806 | 7.809 | 7.835 | 7.902 | 7.923 |
| 1.2 | 1.3802 | 7.868 | 7.916 | 7.965 | 7.883 | 8.134 | 8.124 | 8.163 | 8.211 | 8.236 |
| Intercept | | 5.803 | 6.212 | 6.467 | 6.334 | 6.706 | 6.715 | 6.768 | 6.985 | 6.876 |
| Slope | | 1.525 | 1.267 | 1.086 | 1.114 | 1.029 | 1.017 | 0.998 | 0.862 | 0.973 |
| RSQ | | 0.98 | 0.99 | 0.99 | 1.00 | 1.00 | 1.00 | 1.00 | 0.99 | 1.00 |

**Table S3. 7F conjugate intact monosaccharide concentrations and conjugation levels calculated from RP-UPLC analysis.**

| **Monosaccharide** | **Gal** | **Glc** | **Rha** | **GlcNac** | **GalNac** |
| --- | --- | --- | --- | --- | --- |
| FLR peak area | 5600524 | 10066691 | 29192871 | 28818565 | 25135017 |
| Monosaccharide in Rx (µg) | 0.208 | 0.156 | 0.250 | 0.178 | 0.173 |
| Dilution factor | 500 | 500 | 500 | 500 | 500 |
| Sample [Monosaccharide] (µg/mL) | 104 | 77.8 | 124.9 | 89.0 | 86.4 |
| Monosaccharide Mw (Da) | 180 | 180 | 164 | 221 | 221 |
| [Monosaccahride] (mM) | 0.578 | 0.432 | 0.761 | 0.403 | 0.391 |
| Monosaccharide unit normalized against GalNac (one unit) | 1.48 | 1.11 | 1.95 | 1.03 | 1.00 |
| Theoretical monosaccharide unit in 7F a repeating unit | 2 | 1 | 2 | 1 | 1 |
| % inatct (unconjugated) saccharide | 74 | 111 | 97 | 103 | 100 |
| % conjugation per saccharide (conjugation level) | 26 | 0 | 3 | 0 | 0 |
